# Tumor-associated macrophages in the tumor immune microenvironment of non-small cell lung cancer

**DOI:** 10.3389/fimmu.2026.1738437

**Published:** 2026-10-02

**Authors:** Suhaila Fares Albert Saldana, Nada Daiyan Abdulrab, Afsheen Raza

**Affiliations:** 1 Department of Biomedical Sciences, College of Health Sciences, Abu Dhabi University, Abu Dhabi, United Arab Emirates; 2 Cancer Research Institute, Office of Research and Sponsored Programs, Abu Dhabi University, Abu Dhabi, United Arab Emirates

**Keywords:** immunotherapy resistance, macrophage reprogramming, microRNA, non-small cell lung cancer, tumor microenvironment, tumor-associated macrophages

## Abstract

**Background:**

Non-small cell lung cancer (NSCLC) remains the leading cause of cancer mortality, and resistance to immune checkpoint inhibitors (ICIs) is largely driven by the tumor immune microenvironment (TIME), in which tumor-associated macrophages (TAMs) are major regulators.

**Objectives:**

This review summarizes recent advances in understanding TAM heterogeneity, their contribution to immune resistance in NSCLC, and emerging therapeutic strategies targeting TAMs.

**Methods:**

A literature search was conducted using PubMed and Google Scholar, focusing on studies published between 2021 and 2025. Key search terms included “non-small cell lung cancer”, “tumor immune microenvironment”, “macrophage heterogeneity”, “M1 macrophages”, “M2 macrophages”, “SPP1+ macrophages”, “TREM2+ macrophages”, “immune checkpoint inhibitor resistance”, “macrophage metabolic reprogramming”, and “TAM-targeted therapies”. Relevant studies addressing TAM heterogeneity, immune resistance, metabolic and molecular regulation, and therapeutic targeting in NSCLC were prioritized, while earlier studies were included where necessary to provide mechanistic context.

**Results:**

Recent evidence demonstrates that TAMs comprise diverse functional subsets rather than a simple M1/M2 duality. Distinct populations including CXCL9+, SPP1+, C1Q+, and TREM2+ macrophages differentially regulate T-cell exclusion, immune checkpoint resistance, angiogenesis, and metabolic remodeling. Therapeutic approaches targeting CSF1R, CD47-SIRPα, metabolic pathways, nanoparticles, epigenetic regulators, engineered macrophages, and microRNA-based strategies show promise but remain limited by macrophage plasticity and compensatory immune mechanisms.

**Conclusion:**

A comprehensive understanding of TAM heterogeneity within the tumor immune microenvironment provides a framework for overcoming immune resistance in NSCLC. Future therapeutic strategies should integrate biomarker-guided patient selection with combination approaches that simultaneously target macrophage plasticity and immune suppression.

## 1 Introduction

Lung cancer is the leading cause of cancer mortality worldwide, with non-small cell lung cancer (NSCLC) accounting for approximately 85% of all cases (1). While the advent of immune checkpoint inhibitors (ICIs) has profoundly altered clinical outcomes in patients with advanced NSCLC, most patients are non-responsive or eventually develop resistance to therapy. This highlights the central role of the tumor immune microenvironment (TIME) in regulating the immune response and treatment effectiveness (2). The histologic subtypes of NSCLC show different immune and stromal environments that affect tumor-associated macrophage (TAM) composition and function. Lung adenocarcinoma (LUAD) often has more immune diversity and a higher presence of metabolically altered and secreted phosphoprotein 1 (SPP1+) macrophages, especially in low-oxygen tumor areas. In contrast, lung squamous cell carcinoma (LUSC) typically has more myeloid cells and notable inflammatory signals, with significant immune suppression caused by cytokines and immune checkpoint ligands from TAMs. These differences indicate that TAM-related immune suppression and treatment weaknesses vary across NSCLC and might need specific approaches for intervention (3).

The tumor microenvironment (TME) consists of a network of cancer cells, stromal fibroblasts, endothelial cells, and a complex of immune infiltrate, in which TAMs form a marginalized and flexible subpopulation (4). Within the TME, the tumor immune microenvironment (TIME) comprises infiltrating immune cells and their interactions, playing a central role in regulating immune surveillance, immune evasion, and responses to immunotherapy in NSCLC. Accumulating evidence indicates that TAMs comprise highly heterogeneous, plastic, and diverse populations. This means that macrophages can acquire various phenotypes depending on their environmental stimuli, where different populations and components of the TIME exist, simultaneously exhibiting properties of both M1 and M2 polarized states (5–7). M1-like macrophages are inflammatory cells that regulate immune responses and inhibit tumor growth and metastasis. On the other hand, M2-like macrophages are anti-inflammatory; they participate in wound healing and have poor antigen-presenting capabilities (4). M2-like TAMs tend to accumulate in NSCLC tumors, playing an important role in immunotherapy resistance by reducing T cell influx and inhibiting antigen presentation (8). TAMs play a pivotal role in immunosuppressive TME; their modulation can be targeted as a therapeutic strategy. This review first examines the heterogeneity and functional plasticity of TAMs within the tumor immune microenvironment of NSCLC. It then discusses how distinct TAM subsets contribute to immune evasion and resistance to immune checkpoint inhibitors before reviewing current therapeutic strategies aimed at depleting, reprogramming, or functionally targeting TAMs to improve immunotherapy outcomes.

## 2 Tumor-associated macrophage heterogeneity and its role in immune resistance

Tumor-associated macrophages (TAMs) in NSCLC consist of heterogenous groups influenced by the cell’s origin, location, metabolic conditions, and tumor-derived signals. Instead of fitting into a strict M1/M2 classification, these TAMs exist on a spectrum of inflammatory and immunosuppressive states. Distinct subsets have been identified through single-cell and spatial analyses, such as those that are CXCL9+/CXCL10+, C1Q+, SPP1+, and TERM2+, and the way in which these subsets are distributed and function affects tumor progression, immune evasion, and ICIs response.

### 2.1 Cellular origin of TAMs in NSCLC

In NSCLC, two main sources lead to different populations of TAMs. Tissue-resident alveolar macrophages (TRAMs) come from embryonic precursors. They occupy the alveolar regions and have a high presence of lipid-metabolism programs, such as PPARG signaling. While TRAMs usually help maintain balance in the body, they can develop immunosuppressive traits when influenced by tumor-related factors such as transforming growth factor β (TGF-β) and granulocyte-macrophage colony-stimulating factor (GM-CSF) (6).

On the other hand, monocyte-derived macrophages (MDMs) are drawn from the bloodstream mainly through CCL2-CCR2 and CCL5-CCR5 signals. They undergo changes within the tumor microenvironment. Depending on various local signals, such as low oxygen, lactate buildup, and cytokines from cancer cells, MDMs can transform into inflammatory CXCL9^+^ macrophages or become highly immunosuppressive SPP1^+^ types. Notably, lineage-tracing and transcriptional studies show that MDMs mainly create SPP1^+^ and C1Q^+^ TAM states, which are both strongly linked to resistance against immune-checkpoint inhibitors in NSCLC (5).

### 2.2 Spatial heterogeneity of TAMs

Spatial transcriptomics has shown that TAM phenotypes in NSCLC are greatly influenced by their microanatomical niches. Distinct macrophage subsets localize to hypoxic cores, perivascular regions, and invasive margins. Hypoxic tumor zones are rich in SPP1^+^ and Vascular Endothelial Growth Factor A (VEGFA^+^) TAMs, where Hypoxia-Inducible Factor 1-Alpha (HIF-1α)-driven metabolic changes support glycolysis, angiogenesis, and remodelling of the extracellular matrix. In contrast, perivascular areas house LYVE1^+^/MRC1^+^ perivascular macrophages that maintain blood vessel integrity and help tumor cells enter the bloodstream. At the invasive tumor margins, CXCL9^+^/CXCL10^+^ inflammatory TAMs accumulate, attracting CXCR3^+^ effector T cells and linking to better responses to immune-checkpoint inhibitors (6, 9).

### 2.3 Single-cell and transcriptional heterogeneity of TAMs

Single-cell RNA sequencing (scRNA-seq) of human NSCLC samples demonstrates mixed inflammatory and suppressive characteristics of macrophages driven by signals from the tumor. Pro-inflammatory CXCL9^+^/CXCL10^+^ TAMs, often linked to interferon (IFN)-stimulated pathways, coexist with immunosuppressive C1Q^+^, SPPI^+^, or FN1^+^ TAMs (6). This spectrum helps explain why some therapies targeting TAMs do not succeed; focusing only on M2-like macrophages does not address all immunosuppressive states.

Integrative single-cell and spatial omics studies from 2021 to 2025 have further identified a range of TAM states, including CXCL9^+^, SPP1^+^, C1Q^+^, LAM/TREM2^+^, and proliferating macrophages. CXCL9^+^ TAMs are linked to increased sensitivity to PD-1/PD-L1 blockade, while SPP1^+^ and LAM/TREM2^+^ groups are associated with resistance to immune checkpoint inhibitors and worse outcomes. These findings highlight that the distribution of TAMs, rather than their overall numbers, is a key factor in determining therapeutic responses (6).

### 2.4 SPP1^+^ tumor-associated macrophages in NSCLC

Secreted phosphoprotein 1 (SPP1), also known as osteopontin, is a secreted phosphorylated glycoprotein expressed by tumor cells, macrophages, and stromal cells. SPP1 promotes tumor progression through extracellular matrix remodeling, epithelial-mesenchymal transition (EMT), metabolic reprogramming, and activation of the PI3K/Akt and MAPK pathways. In addition, SPP1 regulates immune cell infiltration and function by promoting M2 macrophage polarization, suppressing CD8+ T-cell activity, and modulating immune checkpoint pathways, including programmed cell death protein 1 (PD-1) and cytotoxic T-lymphocyte-associated protein 4 (CTLA-4). In NSCLC, particularly lung adenocarcinoma (LUAD), elevated SPP1 expression is associated with an immunosuppressive TIME, resistance to immunotherapy, and poor clinical outcomes (6, 10–14).

SPP1 produced by NSCLC tumor cells and TAMs promotes M2 polarization, induces vascular endothelial growth factor (VEGF) expression, and promotes tumor progression. SPP1+ TAMs contribute to immune evasion by suppressing CD8+ T-cell function through the CD39/CD73/A2AR axis, upregulating programmed death-ligand 1 (PD-L1), and interacting with CD44 and integrin receptors on T cells. These findings identify SPP1 as a promising therapeutic target for restoring antitumor immunity and improving immunotherapy responses in NSCLC (6, 15, 16).

ScRNA-seq performed on lung tissues identified a macrophage cluster, Macrophage-C19-SPP1. This cluster exhibited strong interactions with myeloid and T cells. It was significantly enriched in metastatic tissues, specifically brain metastases, where they were enriched with myeloid cells and had reduced T cells, compared to normal lung or primary tumor sites. Analysis of gene expression in SPP1+ TAMs also revealed a reduction of MHC class I and II genes, indicating impaired antigen presentation functions, as well as elevated M2 macrophage markers (CD163, ARG1) and immunosuppressive cytokines (IL-6, IL-10), all contributing to the immunosuppressive environment and poor prognosis (16).

Matsubara et al. (2022) revealed that SPP1 was strongly expressed by MDMs, making it a potential marker to distinguish between TRMs and MDMs. Double immunohistochemistry was used to evaluate SPP1 expression in both cancer cells and TAMs separately. SPP1 expression in TAMs predicted poor prognosis in LUAD, whereas it did not correlate with outcomes for cancer cells. This is because SPP1 expression is dependent on the macrophage differentiation state. Specifically, GM-CSF-mediated macrophage differentiation drives high SPP1 production in MDMs (10, 14).

### 2.5 M1 macrophages: anti-tumor phenotype

Among the heterogeneous TAM populations identified in NSCLC, macrophages can be broadly categorized into inflammatory M1-like and immunosuppressive M2-like phenotypes. Although this classification oversimplifies the continuum of macrophage states, it remains a useful framework for understanding their contrasting roles in anti-tumor immunity and immune resistance.

M1 macrophages are at the inflammatory end of the TAM; they show strong responses to interferon gamma (IFN-γ), Toll-like receptor (TLR) ligands, and STAT1/NF-κB signaling. In NSCLC, these M1 macrophages, especially the CXCL9^+^ and CXCL10^+^ subsets, are crucial for maintaining antitumor immunity. Their presence is associated with better infiltration and activation of CD8^+^ T cells, leading to favorable outcomes during immune-checkpoint blockade (6).

Single-cell RNA-sequencing studies of NSCLC tumors have identified distinct clusters of M1-like TAMs. These are characterized by high levels of CXCL9^+^, CXCL10, CXCL11, GBP1-5, IRF1, STAT1, IDO1, and antigen-presentation molecules such as CD74 and HLA-DRA. These transcriptional features indicate a greater ability to present antigens and produce chemokines that attract CXCR3^+^ CD8^+^ T cells. Spatial transcriptomics shows that CXCL9^+^ TAMs tend to gather at the edges of invasive tumors, where T-cell priming and immune activation occur most intensively (6).

Recent studies indicate that interferon-related M1 signatures are strongly linked to clinical benefits from immune-checkpoint inhibitors. Genes such as CXCL9^+^, CXCL10, IDO1, STAT1, IL2RB, and CD8A were significantly upregulated in patients who responded to PD-1/PD-L1 therapy compared to those who did not (17). Mechanistically, IFN-γ signalling boosts antigen presentation by increasing HLA-II molecules, enhances the recruitment of cytotoxic T cells through potent chemokines, and stimulates macrophages to produce nitric oxide and reactive oxygen species. Tumors rich in M1-like TAMs, therefore, show higher immunogenicity and sensitivity to ICI therapy (17).

In the broader context of TAM diversity, M1 macrophages are nvot fixed entities but instead occupy the inflammatory end of a dynamic continuum. CXCL9 TAMs often come from monocyte-derived macrophages in IFN-rich environments. However, maintaining these cells is challenging due to suppressive signals in TIME. Factors such as hypoxia and lactic acid accumulation hinder M1 polarization through HIF-1α-dependent pathways. Additionally, immunosuppressive subsets such as SPP1^+^ and C1Q^+^ TAMs tend to predominate in ICI-resistant tumors. Therefore, keeping M1-like states alive requires modulating the surrounding microenvironment, not just directly stimulating macrophages (8).

Several treatment strategies are being developed to boost M1 macrophage polarization in NSCLC. TLR agonists such as CpG and R848 activate NF-κB signalling and lead to increased production of inflammatory cytokines, pushing macrophages toward an M1-like state (18). Other agents that enhance IFN-γ-STAT1 signalling also support M1 transcriptional programs; furthermore, targeting pathways of lactate and adenosine shows promise in reversing metabolic suppression of M1 differentiation (5). Despite these advancements, the NSCLC microenvironment tends to exert strong counter-regulatory effects, including IL-10 secretion, PGE2 production, and rewiring from hypoxia, which quickly push macrophages back to immunosuppressive states. Thus, achieving stable M1 polarization remains a major therapeutic challenge (8, 19, 20).

### 2.6 M2 macrophages: pro-tumor phenotype

Within the TIME, TAMs frequently acquire M2-like characteristics associated with anti0inflammatory immune responses. These macrophages support tumor survival, progression, and metastasis through multiple immunosuppressives and pro-tumor mechanisms (21). Cytokines such as IL-4, IL-10, CSF1, and TGF-β, as well as hypoxic conditions commonly found in the TME, drive the differentiation of macrophages towards an M2-like state. These M2-like TAMs express characteristic markers, including CD163, CD206, and arginase-1 (ARG1), and secrete anti-inflammatory cytokines, such as IL-10 and TGF-β, which suppress the proliferative effect of effector T cells and promote tumor immune evasion (4).

Additionally, M2-like TAMs contribute to tumor growth through angiogenesis, extracellular matrix (ECM) remodeling, epithelial-mesenchymal transition (EMT), and metastasis (4). In patients with NSCLC, elevated M2-like TAM infiltrates are associated with resistance to immunotherapy, particularly against PD-1/PD-L1 checkpoint inhibitors, and correlates with poor clinical outcomes (22). Mechanistically, these macrophages modulate immune responses by expressing immune checkpoint ligands, releasing immunosuppressive cytokines, and altering the function of dendritic cells and cytotoxic T lymphocytes (8).

Immunohistochemical analyses of NSCLC tissue further demonstrate that increased stromal M2-like TAM density is associated with poor tumor differentiation, proliferation, invasive size, lymph node metastasis, pathological stage, and poor prognosis. Notably, C-reactive protein (CRP) levels and Ki-67 proliferation index were also significantly high in stromal M2-high tumors (20).

#### 2.6.1 Metabolic landscape of M2 macrophages

In addition to cytokine signalling, metabolic reprogramming is a key driver of TAM polarization within the TME. Alterations in glucose, lipid, and amino acid metabolism promote the development of immunosuppressive M2-like TAMs and contribute to tumor progression, immune evasion and resistance to therapy (5, 7, 23). Metabolically, M2 macrophages favour oxidative phosphorylation (OXPHOS) and fatty acid oxidation (FAO), both of which are upregulated by the presence of lactate and adenosine due to tumor accumulation. Increased levels of FAO in M2 macrophages provide a constant energy supply, fuel the tricarboxylic acid (TCA) cycle, and increase OXPHOS. These processes contribute to the resolution of inflammation (24, 25). Lactate, a major byproduct of tumor glycolysis, promotes M2-like polarization by activating hypoxia-inducible factor 1 alpha (HIF-1α), leading to increased expression of vascular endothelial growth factor (VEGF) and arginase-1 (ARG1), thereby enhancing angiogenesis, immunosuppression, and tumor progression (5, 23, 26). Hypoxia also promotes extracellular adenosine accumulation through ATP degradation. Adenosine signalling via A2AR and A2BR receptors on macrophages induces M2-like polarization, suppresses T-cell and natural killer (NK) cell activity, and promotes immune escape. Furthermore, adenosine upregulates CD39 and CD73 expression, reinforcing an immunosuppressive TME. Preclinical studies have shown that inhibition of A2AR signalling reduces tumor growth and metastasis, highlighting this pathway as a promising therapeutic target (26–28).

Additionally, M2 macrophages show increased metabolism of glutamate, glutamine, and L-arginine, which helps maintain immunosuppression and promotes tumor growth (4). Collectively, these metabolic alterations reinforce the immunosuppressive phenotype of TAMs and represent promising therapeutic targets (25).

The polarization of TAMs to the M2 phenotype is also regulated by the Peroxisome Proliferator-Activated Receptor (PPAR) in the lipid metabolism pathway. Lipid metabolism also regulates TAM polarization through the peroxisome proliferator-activated receptor (PPAR) family. Among the three isoforms (PPARα, PPARγ, and PPARβ/δ), PPARγ plays a dominant role in promoting M2 polarization by enhancing fatty acid oxidation, oxidative phosphorylation, and glutamine metabolism, thereby supporting the transcription of M2-associated genes (7, 29–31). Fatty acids are taken up through the scavenger receptor CD36 with the assistance of fatty acid-binding proteins (FABPs), resulting in intracellular lipid droplet accumulation that sustains M2 macrophage function. High expression of CD36, PPARγ, and FABPs in M2-like TAMs enhances fatty acid uptake, promotes oxidative metabolism, and reinforces immunosuppressive activity within the TME. In addition, TAMs compete with other immune cells for lipid nutrients, further impairing antitumor immunity (7, 26, 32).

#### 2.6.2 Lipid-associated TAMs

Another key subset is lipid-associated macrophages (LAMs), defined by their expression of TREM2, APOE, LIPA, and FABP5. They appear in response to oxidative stress and tissue death. These cells show increased fatty acid oxidation, lower antigen presentation, reduced IFN signalling, and significant tumor-promoting activity, similar to TREM2^+^ suppressive macrophages found in various solid tumors (24).

Lipid-associated TAMs are characterized by the expression of lipid-associated gene signatures, including APOC1, APOE, ACP5, and FABP5. These TAMs show an enrichment of lipid metabolism and OXPHOS pathways, regulated by the transcription factor MAF. They are reportedly found in multiple cancers, including NSCLC (33). In EGFR-mutant NSCLC, a subtype of macrophages has been linked to the TKI osimertinib resistance and leptomeningeal metastases (LMs) development. Using scRNA-seq of CSF, researchers identified a population of lipid-associated macrophages (LAMs) known as RNASE1_M. This subtype exhibited gene expression profiles associated with lipid metabolism and phagocytosis, including APOC1, APOE, TREM2, FABP5, LGALS3, GPNMB, and PLA2G7, as well as subtype markers ALDH1A, RNASE1, MAFB, and MITF. Additionally, RNASE1_M was found to be regulated by Midkine (MDK), which is expressed at significantly higher levels in tumor tissues compared to normal tissues in LUAD and SCC. Macrophage phenotype changes were observed in LMs, characterized by enhanced immunosuppression and an M2 polarized phenotype. The expression of MDK facilitates immune escape associated with CD47-SIRPA expression, contributing to T cell exhaustion and immunosuppression, leading to tumor progression and resistance (34).

#### 2.6.3 TREM2+ tumor-associated macrophages

Studies have identified a TREM2+ TAM subset in NSCLC, which accumulates in tumor tissues rather than in peritumor tissues. Increased TREM2+ TAM infiltration was associated with higher grade tumor, advanced tumor growth, and lymph metastasis. TREM2+ TAMs exhibit features of both M1 and M2-like macrophages. They show increased expression of apolipoprotein genes, including APOC1, APOC2, and APOE, together with SPP1, C1QC, MAF, and MAFB. Fatty acid metabolism pathways and protumor pathways were also enriched in TREM2+ TAMs, while immunoresponsive and DNA repair pathways were significantly downregulated. In the TME of NSCLC, TREM2+ TAMs contribute to the immunosuppressive environment by influencing the immune cell landscape. Increased expression of TREM2+ TAMs was associated with a decrease in naïve B cells and plasma cells. Meanwhile, immune checkpoints PDCD1, CD274, CTLA4, LAG3, HAVCR2, TIGIT, and ENTPD1, as well as cytokines IL10 and TGFB1, were increased at the mRNA level. The study suggests that TREM2+ TAMs do not specifically influence the infiltration or percentage of CD8+ T cells. Instead, they may mediate the dysfunction of CD8+ T cells through their interactions with Tregs. Additionally, enrichment of negative regulation of T cell-mediated immunity and exhaustion of CD8+ T cells was observed in TCGA-LUAD samples with high levels of TREM2+ TAMs (35).

TREM2+ TAMs infiltration can serve as a predictor of poor clinical outcomes and prognosis in patients with NSCLC. High levels of TREM2+ TAM are correlated with worse prognosis, shorter overall survival, and reduced recurrence-free survival. Additionally, TREM2+ TAMs appear to be linked to genomic alteration status in NSCLC, particularly with EGFR mutations. Tumor samples from LUAD patients with EGFR mutations show significantly higher levels of TREM2+ TAMs compared to non-mutated tissues. Given that EGFR mutations are associated with a low response rate to ICB, researchers investigated the significance of TREM2+ TAMs in the responsiveness to ICB in NSCLC. Patients with high TREM2+ TAMs infiltration experienced tumor progression following PD-L1 blockade. TREM2+ TAM-high patients reported an ORR of 14.29%, compared to 31.58% in TREM2+ TAM-low patients. These findings offer a potential therapeutic strategy to reprogram the immunosuppressive TME by targeting TREM2+ TAMs and enhancing response to ICB in NSCLC patients (35).

#### 2.6.4 Other regulators and biomarkers of M2 polarization

Studies reveal other regulators of M2 polarization, for instance, Immunoglobulin-like transcript (ILT) 5, a negative regulator of immune response and activation, expressed in dendritic cells, monocytes, macrophages, and granulocytes. The overexpression of ILT5 can shift macrophages toward an M2-like phenotype, resulting in enhanced immune evasion and reduced T-cell cytotoxicity (8, 22). JAK2/STAT3 signalling pathway is shown to be changed in the M2 polarization state, and its functional interference HM13 knockdown, which greatly inhibits metastasis, switches the polarization to favour an antitumor response (36, 37). The activation of the JAK2/STAT3 signalling pathway promotes the polarization of M2 macrophages by inhibiting anti-tumor cytokines like IL-1β, TNF-α, and IL-18, while activating pro-tumor cytokines like IL-6, IL-10, and VEGF. Negative regulators of the JAK2/STAT3 signalling pathway, like suppressors of cytokine signalling (SOCS), protein inhibitors of activated STATs (PIAS), and protein tyrosine phosphatases (PTP), may influence the direction of macrophage polarization and offer potential therapeutic targets (38).

Gene-level biomarkers, such as MRO, are also described as prognostic indicators of M2 macrophage status in NSCLC, indicating that gene-level biomarkers can be used to detect immune infiltration and treatment responsiveness (39, 40). Additionally, 114 M2 macrophage-related genes were identified, including ABHD5, HS3ST2, TM6SF1, CAPZA2, LEPROT, HNMT, and MRO. These genes were associated with a higher M2 infiltration in NSCLC tissue and demonstrated great predictive performance in patients. Notably, MRO may represent a risk factor that has not previously been reported in NSCLC, highlighting its potential as a significant risk factor (41).

Collectively, these findings demonstrate that TAM heterogeneity extends beyond the traditional M1/M2 classification and is driven by complex molecular, metabolic, and epigenetic mechanisms. Immunosuppressive TAM subsets, particularly M2-like, SPP1+, and TREM2+ macrophages, play central roles in shaping the TIME by suppressing cytotoxic T-cell activity, promoting immune evasion, and contributing to resistance to ICIs. Consequently, these diverse TAM populations have emerged as promising therapeutic targets, promoting the development of strategies aimed at depleting, reprogramming, or functionally modulating macrophages to restore effective antitumor immunity in NSCLC.

## 3 Tumor-associated macrophage-targeted therapeutic strategies in NSCLC

Given the central role of TAM heterogeneity in driving immune suppression and resistance to ICIs, considerable efforts have focused on developing therapeutic strategies that either deplete immunosuppressive TAMs or reprogram them toward an antitumor phenotype. Current TAM-targeted approaches in NSCLC include CSF1-CSF1R axis inhibition, CD47-SIRPα blockade, nanoparticle-mediated TAM reprogramming, epigenetic modulation, miRNA-based reprogramming, combination therapies, and engineered macrophages for drug delivery (**Table 1**; **Figure 1**).

**Table 1:** TAM-targeted therapies in NSCLC.

Therapy/strategy	Class	Agent	Target	Mechanism	Phase	Cohort	Efficacy/response rate	Key toxicities	Limitations	Reference
CSF1/CSF-1R blockade	Small-molecule inhibitor	Pexidartinib (PLX3397) + Pembrolizumab (anti-PD-1 mAb)	CSF-1R (and cKit/Flt3	Blocks TAM recruitment with a combined immune suppression blockade with PD-1 inhibition	Phase 1/2a	Advanced solid tumors, including NSCLC.	Limited evidence of clinical efficiency; no NSCLC-specific ORR reported.	Not reported; generally well tolerated-, associated with AST/ALT liver enzyme elevation, fatigue, nausea, etc.	Not reported. CSF-1R blockade is insufficient; compensatory myeloid pathways	(42)
	Small molecule inhibitor	ARRY-382 + Pembrolizumab (anti-PD-1 mAb)	CSF-1R	Decreases TAMs and MDSCs, and reprograms TAMs in the TME to increase antigen presentation and T cell activation.	Phase 1b/2	Advanced solid tumors, including NSCLC.	Overall, limited ORR; no specific NSCLC ORR reported. Phase 1b ORR 10.5%. Phase 2 ORR 3.7% (PDA only); 0.0% (PD-1/PD-L1 IR and prOVCA cohorts.	Safety profile is generally tolerated and well-manageable. Phase 1b DLTs in 15.8. AEs in 94.6%. common toxicities: AST/A LT/CPK/Amylase/Lipase elevations, fatigue, pruritus, Rash, and nausea. Discontinuations 10.5%. No treatment-related death. PFS 1.4-4.7 months.	On-target CSF-1R modulation, but limited clinical benefit	(43)
	Small molecule EGFR-TKI	Pexidartinib + Osimertinib	CSF-1R + EGFR	EGFR-TKI induces CD8+ T-cell immunity, combined with CSF-1R inhibitor, suppresses M2 (CD206^+^) macrophages, and enhances anti-tumor immunity.	Preclinical mouse model.	EGFR-mutated lung cancer cells.	Osimertinib response peaks ~14 days; residual persister cells. Pexidartinib reduces CD206^+^ TAMs and enhances antitumor efficacy	N/A	Not tested clinically.	(44)
	Anti-CSF-1R mAb	AMG 820 + pembrolizumab (anti-PD-1 mAb)	CSF-1R	Inhibits and depletes TAMs, overcoming anti-PD-1 resistance and enhancing the T cell response with PD-1 inhibition.	Phase 1b/2	Advanced solid tumors, including NSCLC.	Overall limited activity. No phase 2 cohort met efficacy threshold. irPR in 3% of patients, 1/19 (5.3%) NSCLC patients, previously nivolumab-treated, PD-L1 low.	DLTs in 6%. 99.1% AEs in patients; 87.9% grade 3 AEs. Common AEs: AST increase, fatigue, facial/periorbital edema, rash. 13% AMG 820 and 12% pembrolizumab discontinuation due to TRAEs.	Lack of effective depletion or durable reprogramming of intratumoral macrophages, lack of CD68^+^ or CD163^+^ macrophage reduction. Confounding inflammation from PD-1 blockade, adaptive resistance to CSF-1R inhibition lead to Tregs and suppressor myeloid cells activation and recruitment.	(45)
	Anti-CSF-1R mAb	Emactuzumab (RG7155) + Atezolizumab (anti-PD-L1 mAb)	CSF-1R	Depletes immunosuppressive TAMS, with anti-tumor effect exerted.	Phase Ib	Solid tumors, including NSCLC.	Overall ORR 7.7%. For ICB-treated NSCLC, ORR in 5/40 (12.5%) and PFS 2.8 months.	Safe and tolerable, with manageable AEs. No DLTs observed. 47.2% AEs related to study treatment, including periorbital/face edema, fatigue, rash, pruritus, asthenia, anemia, and AST increase	No monotherapy control. Limited TAM depletion and persistent CD163 +TAMs. ICB-treated patients less susceptible to TAM depletion. Inability to distinguish PD-L1 vs CSF-1R–specific effects. Limited profiling of residual TAMs, MDSCs, and CD8+ T cell function. Lack of predictive biomarkers.	(46)
	Anti-CSF-1R mAb	LY3022855 + Durvalumab (anti-PD-L1) or Tremelimumab (anti-CTLA-4)	CSF-1R	Block immunosuppressive TAMs’ survival and recruitment with PD-L1/CTLA-4 blockade to reprogram TAMs in the TME.	Phase 1a/1b	Advanced solid tumors, including NSCLC.	Limited activity. Responses only seen in LY3022855 100 mg + durvalumab 750 mg cohorts (D4A and B2). Overall ORR 4.2%. Overall median PFS 1.87, NSCLC median PFS 1.68 months.	Acceptable tolerability and safety. No DLTs. 87.4% patients had drug-related TRAEs, ~60% grade ≥3 TRAEs. Common AEs: CPK/AST/Lipase increase, hyponatremia, fatigue, and facial edema. No treatment-related death.	Heavily pre-treated, difficult-to-treat population. Limited activity. Unknown resistance mechanisms. Small cohort sizes limit pharmacodynamic and biomarker analyses.	(47)
	CD40 agonist mAb + CSF-1R mAb	Sotigalimab (APX005M) + Cabiralizumab, with or without Nivolumab (anti-PD-1 mAb)	CD40 + CSF-1R	Dual TAM modulation depletes TAMs and activates macrophages and dendritic cells to enhance antigen presentation, restoring T cell activity with Nivolumab.	Phase I	Anti-PD-1/PD-L1-resistant tumors, including NSCLC.	No confirmed ORR or NSCLC-specific ORR. Median PFS ~72 days.	All patients had treatment-related AEs. Most commonly, asymptomatic LDH/AST/ALT/CK increase, and low-grade CRS/infusion reactions. 1 DLT.	Low clinical response rates. Small NSCLC subset, and very limited NSCLC numbers.	(48)
CD47 – SIRPα blockade-	Anti-CD47 mAb	Magrolimab (Hu5F9-G4) + docetaxel (chemotherapy)	CD47	Promotes macrophage phagocytosis of tumor cells, with chemotherapy synergy enhancing phagocytic signals.	Phase 2	Previously-treated metastatic NSCLC	ORR 19.4%. Median PFs 5.4%.	No DLTs. Common ≥3 TRAEs: neutropenia, anemia, leukopenia, asthenia. Discontinuation 19%. No treatment-related deaths.	Not reported. Single-arm phase 2 study with a small NSCLC cohort.	(49)
	CD47 blocker (fusion protein)	Evorpacept (ALX148) + standard anti-cancer antibodies and chemotherapy (pembrolizumab, trastuzumab, ramucirumab, paclitaxel, cisplatin).	CD47	Promotes macrophage phagocytosis while enhancing the activity of anti-cancer antibodies and checkpoint inhibitors.	Phase I	Advanced solid tumors, including NSCLC (CPI resistant/refractory or PD-L1 <50%)	1/23 PR observed in NSCLC	59.5% low grade TRAEs: fatigue, AST/ALT increase, anemia, and platelet decrease.	Not reported. Survival outcomes not defined. Early phase, small NSCLC cohort.	(50)
TLR agonist	TLR7/8 agonist	R848 (with chemotherapy or nanoparticles)	TLR78	R848 activates TLR immunity, stimulating DCs, NK cells, and CD8+ T cells. Membrane-coated nanoparticles enhance interaction with the TME	Preclinical	Murine lung cancer models	Reduced lung tumor growth and metastasis. Prolonged survival, R848-loaded PNP enhanced local retention.	No toxicity observed.	No clinical translation. Risk of systemic inflammation. Dependent on host TLR7 expression. Antitumor effect depends on DCs, CD8+ T cells, and NK cells activation; no direct impact on tumor cells.	(51, 52)
TAMs reprogramming with nanoparticles	TLR agonist-loaded nanocapsules (NC)	Mannose-HA-protamine-NCs loaded with poly(I:C)+R848	TLR3 (poly(I:C)), TLR7/8 (R848) in macrophages	Polymeric nanocapsules deliver dual TLR agonists to TAMs inducing M1 polarization with higher CD86 and lower CD206/Arg1 expression, boost chemokine secretion (CXCL10, CCL5), reduce tumor growth & lung metastasis	Preclinical	Murine lung cancer models (CMT167 orthotopic lung cancer; MN/MCA1 fibrosarcoma lung metastasis)	Significant reduction in lung tumors, systemic activation of antitumor immunity (CXCL10 and IL-6), prevention of spontaneous metastasis, reprogramming of interstitial macrophages.	No weight loss or systemic toxicity observed.	No clinical translation. No significant changes in T cells demonstrated.	(53)
	siRNA nanoparticle delivery system	Folic acid (FA)-modified, ultrasound-responsive nanocarriers delivering SIRPα siRNA and Fe_3_O_4_ (FA@PFP@Fe3O4@LNB)	Folate Receptor-β (FR-β) on TAMs, SIRPα receptor	Fe3O4 nanoparticles activate interferon-regulatory factor 5(IRF5) pathway to stimulate inflammatory response, transitioning M2-type to M1-type macrophages. Delivering SIRPα siRNA to knock down the SIRPα receptor on macrophages enhances phagocytic ability.	Preclinical	Murine NSCLC (LLC)-tumor-bearing C57BL/6 mice, in vitro RAW264.7 cells.	Inhibition of NSCLC tumor growth, invasion, and metastasis, prolonged mice survival, enhanced intertumoral T cell activation, and increased intertumoral M1 macrophages in vitro and in vivo.	Safe and well-tolerated, no observed systemic toxicity, no significant AEs induced in mice.	No clinical translation, limited ultrasound penetration depth in peripheral lung tissues.	(54)
	miRNA nanoparticle delivery system	CD44 targeting hyaluronic acid-poly(ethylenimine) (HA-PEI)-based nanoparticles encapsulating miR-125.	CD44	miR-125 delivery promotes macrophage differentiation from M2- to M1inflammatory type and enhances anti-tumor effect.	Preclinical	KRAS/p53 mutant GEM NSCLC mouse models	Specific targeting of peritoneal macrophages, migrating to lung areas ablated of macrophages, robust TAM repolarization toward an M1 phenotype, 6-fold increase in M1/M2 ratio, 300-fold increase in iNOS/Arg1 ratio in TAMs	N/A	Preclinical murine data only, no tumor growth, toxicity, survival, or combination efficacy assessed, intraperitoneal administration relied on macrophage migration.	(55)
	Nanoparticle-based dual mAb delivery	Microfluidics-engineered nanovesicles co-delivering anti-CD47 and anti-PD-L1 antibodies	CD47 + PD-L1	Anti-CD47 delivery to block the CD47–SIRPα axis restores TAM phagocytosis, and anti-PD-L1 enhances T-cell activity; co-delivery reduces systemic immunotoxicity and synergistically boosts antitumor immunity.	Preclinical	LLC C57BL/6 mouse models	~2.5-fold increased intratumoral CD47/PD-L1 antibody accumulation, tumor growth inhibition, prolonged mice survival, significant M1/M2 ratio increase, TAM reprogramming.	Good biosafety, reduction of IRAEs, negligible hemolysis, cell toxicity, or on-target off-tumor immunotoxicity.	Preclinical murine data only, new drug remains unevaluated for safety before application in human research.	(56)
Epigenetic modulators	HDAC inhibitor	Vorinostat + Pembrolizumab (anti–PD-1)	HDAC + PD-1	HDACi enhances the response to ICIs, exerting inhibitory effects on MDSCs and inducing the expression of T cell chemokines (e.g. Cxcl9 and Cxcl10) in lung cancer cells, macrophages, and T cells.	Phase 1/1b	Advanced/metastatic NSCLC; both ICI-naïve and ICI-pretreated (refractory or relapsed)	13% PR overall, 6.6% ORR overall in ICI-pretreated. mPFS 2.8-4.6 months in ICI-pretreated, mPFS 7.5 months in ICI-naïve.	Well-tolerated most AEs grade 1-2, 73% TRAEs, common AEs: fatigue, nausea, vomiting, hypothyroidism. No DLTs, no treatment-related deaths, 6% discontinuations.	Limited sample size, limited direct evidence of TAM targeting in patients, no significant depletion of MDSCs.	(57)
	Class I HDAC inhibitor	Entinostat + Pembrolizumab (anti–PD-1)	HDAC + PD-1	HDAC inhibition increases MHC presentation, tumor antigen presentation, and decreases immunosuppressive cells (e.g., MDSC), reducing TAMS, remodeling the TME toward an immune-responsive state.	Phase 1b/2	Recurrent/metastatic NSCLC	Limited efficacy, 9.2% ORR, mPFS 2.8 months, elevated classical circulating monocytes associated with clinical benefit.	82% TRAEs, 58% grade ≥3 TRAEs. Most common AEs: fatigue, diarrhea, anemia, and decreased appetite. No treatment-related deaths	Failure to meet efficacy threshold, heavily pre-treated PD-(L)1 resistant population, outcomes in patients not associated with PD-L1 expression,	(58)

NSCLC, non-small cell lung cancer; CSF-1R, colony-stimulating factor 1 receptor; TAMs, tumor-associated macrophages; TME, tumor microenvironment; MDSCs, myeloid-derived suppressor cells; ORR, objective response rate; DLT, dose-limiting toxicity; AST, aspartate aminotransferase; ALT, alanine aminotransferase; CPK, Creatine Phosphokinase; PFS, progression-free survival; mPFS, median progression-free survival; PDA, pancreatic ductal adenocarcinoma; prOVCA, platinum-resistant ovarian cancer; PD-1, programmed cell death protein 1; PD-L1, programmed death-ligand 1; EGFR, epidermal growth factor receptor; TRAEs, Treatment-related adverse events; irPR, immune-related partial response; mAb, monoclonal antibody; CTLA-4, cytotoxic T-lymphocyte-associated protein 4; CD40, cluster of differentiation 40; CK, creatine kinase; CD47, cluster of differentiation 47; SIRPα, signal regulatory protein alpha; PR, partial response; TLR, toll-like receptor; DCs, dendritic cells; NK cells, natural killer cells; CD8^+^ T cells, cytotoxic T lymphocytes; HA, hyaluronic acid; NCs, nanocapsules; Poly(I:C), polyinosinic–polycytidylic acid; CXCL10, C-X-C motif chemokine ligand 10; CCL5, C-C motif chemokine ligand 5; FA, folic acid; FR-β, folate receptor beta; IRF5, interferon regulatory factor 5; siRNA, small interfering RNA; miRNA, microRNA; HA-PEI, hyaluronic acid–poly(ethylenimine); LLC, Lewis lung carcinoma; KRAS, Kirsten rat sarcoma viral oncogene homolog; GEM, genetically engineered mouse; iNOS, Inducible nitric oxide synthase; Arg1, Arginase 1; IRAEs, immune-related adverse events; HDAC, histone deacetylase; HDACi, histone deacetylase inhibitor; ICIs, immune checkpoint inhibitors; MHC, major histocompatibility complex.

**Figure 1:**
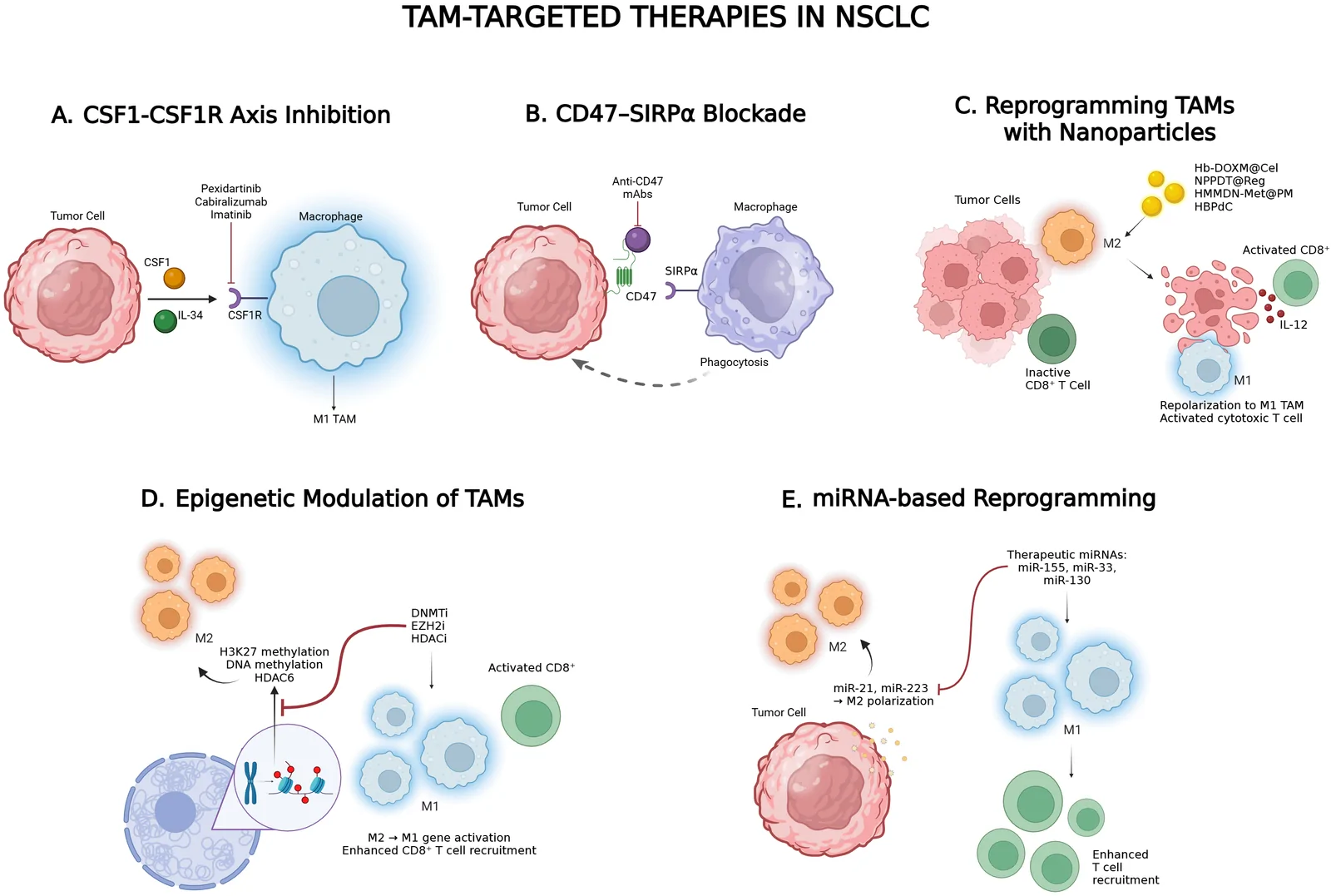
TAM-targeted therapeutic strategies for non-small cell lung cancer (NSCLC). Created with BioRender.com Tumor-associated macrophages (TAMs) play a crucial role in NSCLC progression by promoting tumor growth, angiogenesis, and immune evasion. Several therapeutic strategies have been developed to target TAMs and reprogram their tumor-promoting (M2-like) phenotype toward a tumor-suppressive (M1-like) state: **(A)** CSF1–CSF1R axis inhibition reduces TAM recruitment and survival through blockade of CSF1R signaling using agents such as pexidartinib, cabiralizumab, or imatinib. **(B)** CD47–SIRPα blockade enhances macrophage-mediated phagocytosis of tumor cells by disrupting the “don’t eat me” signal via anti-CD47 monoclonal antibodies. **(C)** Reprogramming TAMs with nanoparticles employ nanocarrier systems (e.g., Hb-DOXM@Cel, NPPD@Reg, HMMDN-Met@PM) to deliver immunomodulatory molecules, inducing M2-to-M1 repolarization and activating cytotoxic CD8^+^ T cells. **(D)** Epigenetic modulation of TAMs targets histone and DNA methylation pathways (e.g., DNMT inhibitors, EZH2 inhibitors, HDAC inhibitors) to activate M1-associated genes and enhance CD8^+^ T-cell recruitment. **(E)** miRNA-based reprogramming uses therapeutic miRNAs (miR-155, miR-33, miR-130) to shift macrophages toward an M1 phenotype while counteracting tumor-derived miRNAs (miR-21, miR-223) that promote M2 polarization.

### 3.1 CSF1-CSF1R axis inhibition

The colony-stimulating factor 1 (CSF-1) and its receptor CSF1R are key regulators of TAM survival, recruitment, and polarization within the NSCLC TME. CSF-1 signalling encourages differentiation into immunosuppressive M2-like macrophage states by activating downstream pathways such as PLC-γ2, STAT3, and ERK1/2. Additionally, IL-34, another CSF1R ligand, supports macrophage recruitment and M2 polarization (19, 59). Higher levels of CSF-1, CSF1R, and IL-34 in NSCLC indicate advanced disease and worse prognosis (19). Therefore, inhibiting CSF1R has been proposed to decrease TAM density and shift macrophage polarization towards more inflammatory phenotypes (4, 21).

Several CSF1R-targeting agents, including small-molecule inhibitors and monoclonal antibodies, have shown promising immunomodulatory effects in preclinical models, but there has been limited success in clinical settings. Early-phase clinical studies have mainly examined CSF1R inhibitors in combination with immune checkpoint inhibitors. In a phase 1b trial, the anti-CSF1R antibody emactuzumab was used alongside the anti-PD-L1 antibody atezolizumab for patients with advanced solid tumors, including NSCLC. Although changes in TAM modulation and increased CD8+ T-cell infiltration were noted, the overall clinical responses were modest. The response rate was low in patients who were resistant to immune-checkpoint therapy, and there was less pronounced TAM depletion in those who had previously received immune checkpoint inhibitors compared to those who had not (46). Similarly, when AMG 820 was combined with pembrolizumab, it showed target engagement but limited anti-tumor effects, achieving an objective response rate of about 3% across advanced solid tumors, including NSCLC. Hepatotoxicity also became a dose-limiting factor (45). Other early-phase combinations, such as LY3022855 with durvalumab or tremelumumab and cabrializumab with APX005M ± nivolumab, showed acceptable safety profiles but minimal objective responses in NSCLC groups, with effectiveness similar to single-agent therapy. Together, these studies reveal several important drawbacks of CSF1R-targeted strategies. First, blocking CSF1R often leads to the compensatory recruitment of other immunosuppressive myeloid cells, including myeloid-derived suppressor cells and neutrophils, which restores immune suppression despite TAM modulation (45, 46, 60–63). Second, CSF1R-independent TAM subsets, especially SPP1+ and TREM2+ macrophages found in hypoxic tumor areas, often remain and continue to support tumor growth. Third, incomplete removal or reprogramming of tissue-resident and hypoxia-adapted TAMs, along with dose-limiting liver toxicity, limits effective drug exposure. Mechanistically, tumors can adapt to CSF1R inhibition by increasing alternative chemokine signaling pathways such as CCL2-CCR2 and CXCL8-CXCR2, which reinstates an immunosuppressive environment and reduces the benefit of combining this with immune checkpoint blockade. These issues likely explain why CSF1R inhibitors show strong preclinical synergy with PD-1/PD-L1 blockade but have not yet resulted in consistent clinical advantages in NSCLC (45, 46).

### 3.2 CD47-SIRPα blockade

CD47 is a cell-surface immune checkpoint molecule that allows tumor cells to escape macrophage-mediated phagocytosis. It does this by engaging signal regulatory protein α (SIRPα), which is found on myeloid cells. In NSCLC, CD47 is often overexpressed and sends a strong “don’t eat me” signal that prevents the innate immune system from clearing the tumor. The co-expression of CD47 and PD-L1 in NSCLC is linked to a poor prognosis, showing that both innate and adaptive tumor immunity are being suppressed (60–62). Disrupting the CD47-SIRPα interaction with monoclonal antibodies or engineered proteins can restore macrophage phagocytosis activity and help clear tumors (30). Preclinical and early clinical studies suggest that blocking CD47 works best when paired with agents that provide pro-phagocytic signals, rather than using it alone. While CD47-targeting methods have had notable success in blood cancers, applying them to solid tumors, such as NSCLC, requires combination approaches (61, 63, 64). Combining CD47 blockade with ICIs or tumor-targeting antibodies has shown synergistic effects. These combinations boost Fc-mediated opsonization and enhance macrophage engagement (63, 65, 66). In NSCLC, early clinical trials of the CD47-blocking antibody magrolimab alongside chemotherapy, such as docetaxel, showed CD47 saturation and signs of clinical activity in heavily treated metastatic patients. However, blood-related toxicities, such as anemia and temporary reticulocytosis, became common dose-limiting side effects, necessitating careful priming and dosing strategies (67). Additional CD47-directed agents, like ALX148, when combined with pembrolizumab, showed partial responses across different tumor types, including NSCLC, with manageable safety profiles in early-phase studies (67).

Despite these progressions, several limitations hinder the effectiveness of CD47-SIRPα blockade in NSCLC. CD47 blockers generally show limited efficacy as monotherapy, without tumor-associated pro-phagocytic signals, such as calreticulin exposure, or Fc-mediated opsonization from combination antibodies (60). Many NSCLC tumors have low baseline pro-phagocytic signaling, which restricts macrophage engagement. Although some cytotoxic agents, including cisplatin, doxorubicin, and EGFR inhibitors, can improve CD47 blockade by increasing pro-phagocytic cues, they also lead to significant on-target blood-related toxicity, notably anemia and thrombocytopenia (68, 69). Therefore, current evidence supports CD47 blockade mainly as a combination-based therapeutic strategy, with ongoing efforts aimed at optimizing partner therapies and dosing schedules to find the right balance between effectiveness and safety (70).

### 3.3 Reprogramming TAMs using nanoparticles

Nanotechnology presents a targeted approach for delivering therapeutic agents specifically to TAMs within the TME. Nanomaterials are utilized to reprogram TAMs to enhance anti-tumor effects and improve the TME overall. Several nanomedicine systems have been developed, including Hb-DOXM@Cel, which is designed to deliver targeted oxygen, chemotherapeutic agents, and immunomodulators to reprogram TAMs and remodel the TME. Here, hemoglobin (Hb) is used as an oxygen carrier, delivering oxygen to the TME, helping to alleviate hypoxia in tumor cells. This helps to reduce M2-like TAM recruitment and polarization, while synergistically releasing Doxorubicin (DOX) to kill M2-like TAMs, and encapsulated drug celecoxib to reprogram macrophages (21, 71).

Another is the NPPDT@Reg delivery system, which delivers the vascular normalizing agent Reglanis (Reg). This helps improve hypoxia and increases oxygen and drug delivery into the tumor through vascular normalization, generating more ROS to kill tumor cells. It also reverses the immunosuppressive environment by reprogramming the TAMs phenotype (21).

HMMDN-Met@PM uses hollow mesoporous manganese dioxide nanoparticles (HMMDN) loaded with metformin (HMMDN-Met), making up the nano-core. It is composed of a macrophage membrane for disguise as an autologous component, modified with M2 macrophage-targeting peptide (M2pep) on its surface to target M2-like TAMs for effective drug delivery. Metformin has been shown to inhibit the mTOR signal and activate the AMPK-NF-κB signal, which inhibits tumor progression and metastasis by enhancing the phosphorylation of MAPK. It also regulates the expression of M1/M2 proportions. However, metformin’s effectiveness is limited by its short life *in vivo* (21, 72).

Resiquimod (R848), a TLR 7/8 agonist, is encapsulated in microparticles (MPs) derived from RAW264.7 macrophage cell lines overexpressing AFP (MPsAFP) that are modified with M2pep (R848@M2pep-MPsAFP). This formulation specifically targets and reprograms M2-like TAMs into an M1-like phenotype. It reprograms these macrophages to present AFP antigens with R848 delivery to CD8+ T cells. This reprogramming alleviates the immunosuppressive TME and activates CD8+ T cell anti-tumor immunity while promoting the differentiation of stem cell-like CD8+ T cells (21, 73). When combined with anti-PD-1 immunotherapy, R848@M2pep-MPsAFP demonstrated a strong synergistic anticancer effect, resulting in a 95.9% inhibition of tumor weight in HCC mice and prolonging survival. Treatment with R848@M2pep-MPsAFP showed strong cytotoxic activity against Hepa1–6 cells expressing alpha-fetoprotein (AFP), but not H22 liver tumors lacking AFP expression. This indicates that M2-like TAMs reprogrammed by R848@M2pep-MPsAFP present AFP antigen to activated CD8+ T cells and specifically target tumors. Building on this principle, the application can be expanded to target lung cancer by using R848@M2pep-MPs derived from RAW264.7 cells that overexpress a tumor-associated antigen, such as Melanoma-associated antigen (MAGE)-A3, overexpressed in NSCLC, to treat lung cancers (73).

M2-like TAMs can be reprogrammed to M1 macrophages by nanoparticles with TLR agonists, STAT inhibitors, or siRNAs. Nanocarriers targeting the mannose receptor on M2-like macrophages deliver mRNA that encodes an M1-like TAMs polarized transcription factor, reprogramming M2-like TAMs for anti-tumor effects. Similarly, micellar nanodrugs co-deliver IKKβ siRNA and a STAT6 inhibitor (AS1517499) to target M2 functions. The siRNA silences IKKβ (involved in activating the NF-κB signaling pathway), while the STAT6 inhibitor prevents the expression of M2 genes. This dual action effectively polarizes TAMs toward M1-type and inhibits tumor growth (74–76).

β-cyclodextrin nanoparticles (CDNPs) deliver immunomodulatory agents such as TLR7/8 agonists to TAMs within the TME *in vivo*. Uptake of CDNPs by the tumor cell releases R848, which acts as an agonist for TLR7 and TLR8 receptors, enabling TAM repolarization and anti-tumor immune response, and synergizing with anti-PD1 immune checkpoint blockade (18, 74). It was demonstrated that CDNP alone accumulated in TAMs but did not elicit an IL-12 response; meanwhile, R848 alone was not able to induce a robust IL-12 response *in vivo*. However, when CDNP and R848 are combined, they successfully induced an IL-12 response and show enhanced nanoparticle uptake by TAMs. This combination increased the delivery of R848 and re-educated macrophages within the TME. Additionally, the combination of CDNP-R848 with anti-PD-1 was found to be synergistic, leading to a reduction in tumor areas. The delivery of R848 to TAMs was also examined in orthotopic lung adenocarcinoma (KP) models. Intravital imaging results indicate that CDNP-mediated delivery resulted in a threefold higher concentration of R848 in TAMs compared to systemically administered, solubilized R848, correlating with the induction of IL-12 response in macrophages (77, 77).

The angiogenic factor VEGF is produced by inflammatory TAMs in lung cancer, leading to the recruitment of macrophages and promoting the proliferation and invasion of cancer cells. To address this issue, researchers developed a nanoparticle system designed with RNAi-M2pep-AuNPs to deliver anti-VEGF siRNA for VEGF silencing in TAMS and lung cancer cells, thereby targeting inflammation. RNAi-M2pep-AuNPs accumulated in lung tumor tissues both *in vitro* and *in vivo*, with uptake by M2 macrophages. Treatment with these NPs demonstrated high specificity, reducing macrophage abundance and recruitment of inflammatory TAMs in lung tumor tissues. The NPs successfully silenced VEGF expression by ~75% in macrophages and ~85% in lung cancer cells. In mice, therapy reduced tumor size by ~95% and increased survival by ~95%. The treatment was well tolerated *in vivo* with no non-specific immune response activation and no toxicity at the administered dose (78).

Furthermore, targeting specificity and bioavailability can be increased by coating nanoparticles with macrophage membranes, thereby reducing systemic toxicity (79). RAW264.7 is a commonly used murine macrophage model, used for anti-tumor drug delivery in cancer therapy. Macrophages possess α4 and β1 integrins that bind to VCAM-1 on cancer cells, allowing them to act as vehicles for delivering therapeutic agents, such as Doxorubicin (DOX), deep into tumor tissue (79, 80). For example, M1 macrophage-biomimetic-targeted nanoparticles (DOX/CAT@PLGA-M1) are designed with a core-shell bilayer membrane, consisting of a polymer (PLGA) that is loaded with catalase (CAT), an oxygen-supplying enzyme, and doxorubicin (DOX), a chemotherapeutic agent, all of which coat the M1 macrophage membrane surface. DOX/CAT@PLGA-M1 showed significantly higher uptake in 4T1 cells, inhibition of tumor growth and metastasis, as well as reduced tumor volume. No significant systemic toxicity or weight loss was observed. The nanoparticles even reduced cardiotoxicity associated with free DOX therapy, exhibiting excellent compatibility, stability, and safety (81).

### 3.4 Epigenetic modulation

Epigenetic changes contribute to the polarization of TAMs by reprogramming macrophages gene expression and function in the TIME. One aspect involves the regulation of reversible mRNA modifications, such as N6-methyladenosine (m6A) modifications, which play an important role in various tumors and immune responses. m6A modifications affect multiple aspects of RNA metabolism and are involved in numerous physiological and pathological processes.

Alterations in key molecules involved in m6A modifications, including METTL3, YTHDF1, YTHDF2, and IGF2BP1, can drive immunosuppression in the TIME, macrophage polarization, or anti-tumor responses (21). Methyltransferase-like 3 (METTL3) is a catalytic enzyme that adds m6A modifications to RNA, impacting RNA stability, translation efficiency, and splicing. In NSCLC, the m6A modification of SRSF2 by METTL3 promotes tumor development. YTHDF2 is responsible for mediating RNA decay, YTHDF1 facilitates mRNA translation, and IGF2BP1 regulates the mRNA translation of many genes, playing a part in promoting NSCLC development through m6 modifications of TKI (82).

The mechanisms by which m6A modifications and METTL3 regulate the TIME are complex and context-dependent. m6A modifications play a role in regulating macrophage activation. Specifically, METTL3 regulates the expression of STAT proteins, which control macrophage polarization. In myeloid cells, METTL3 loss disrupts the m6A-YTHDF2-SPRED2 axis, resulting in enhanced NF-κB activation and increased STAT3 phosphorylation. This contributes to an immunosuppressive environment by upregulating the infiltration of M1/M2-like TAMs. METTL3-mediated regulation of immune cytokine expression may be dependent on the cancer type. In NSCLC/LUAD, enhanced METTL3-mediated m6A modifications increase m6A RNA levels and contribute to M2-associated immunoresistance. METTL3 contributes to a non-inflamed TIME in NSCLC through the upregulation of pro-tumorigenic chemokines, CXCL1, CXCL5, and CCL20, to facilitate the recruitment of immunosuppressive cells such as MDSCs and Tregs. It also affects PD-L1 mRNA stability, thereby decreasing its expression through m6A-mediated modifications. NSCLC patients with lower expression of METTL3 show better prognosis with anti-PD-1 therapy; therefore, targeting METTL3 to reprogram the TIME to be more inflamed may be a good strategy to improve the efficiency of anti-PD-1 therapy in NSCLC patients (83–85).

Bai et al. (2024) demonstrated that TRPV1 overexpression aggravates tumor growth and metastasis in NSCLC and promotes M2 macrophage polarization. TRPV1 is enriched in m6A modification and is positively correlated with METTL3, which is upregulated in LUAD tissues compared to normal tissues. Both METTL3 and IGF2BP1 can regulate TRPV1 expression through m6A methylation modification, promoting cell growth and metastasis in NSCLC. METTL3 knockdown repressed the tumor growth, metastasis, and M2 polarization *in vivo*; however, these effects were weakened by the overexpression of TRPV1. Silencing TRPV1 led to tumor suppression and decreased CD163+ cells in H1299 and A549 cells, which indicates inhibition of M2 macrophage polarization (86).

Alterations in DNA methylation in TAMs influence their immunosuppressive phenotype by affecting the immunological gene expression, contributing to immune evasion and tumor progression. Hypoxia-induced DNA methyltransferases (DNMTs) activity in TAMs lead to hypermethylation of promoter regions at M1-associated genes. This results in the silencing of genes involved in antigen presentation and the production of pro-inflammatory cytokines. Additionally, altered DNA methylation of oxidative metabolism genes has been shown to polarize these macrophages toward an M2-like phenotype. Blocking DNMT activity inhibits the metabolic reprogramming of macrophages, shifting them toward an inflammatory phenotype (87, 88).

The suppressive phenotype of TAMS is enhanced by histone methyltransferases and deacetylation. Enhancer of zeste homolog 2 (EZH2) is a histone methyltransferase and a key subunit of the polycomb repressive complex 2 (PRC2), which modifies chromatin structure. EZH2 plays a crucial role in silencing tumor suppressor genes through hypermethylation and promoting the overexpression of oncogenes by hypomethylation, thereby promoting the proliferation and metastasis of lung cancer (48). EZH2 may also inhibit the anti-tumor effects of macrophages and NK cells by reducing the expression of CXCL10 and CCL2 chemokines. 10.3390/cells11243958 Additionally, PRC2, along with H3K27me3 and EZH2-mediated DNMT1, reduces the expression of Th1-type chemokines CXCL9 and CXCL10, a mechanism associated with immune evasion. In lung cancer, the EZH2 and DNMT1 inhibition restores CCL2 expression, which induces the infiltration of M1 macrophages (87).

Methylation of histone H3 lysine-27 (H3K27) epigenetically regulates M2 polarization. In the TME, IL-4 and IL-10 induce the expression of H3K27 demethylase Jumonji domain-containing 3 (JMJD3) in TAMs. This decreases H3K27 methylation/repression of M2 genes (e.g., ARG1 and CHI3L1) and genes that inhibit inflammation (e.g., IL1RA), leading to the transcriptional activation of M2 macrophage marker genes (88, 89).

HDAC6 is a histone deacetylase (HDAC) that possesses two catalytic sites and exhibits broad substrate specificity, allowing it to deacetylate a diverse range of substrates. It is overexpressed in many cancers, including LUAD, and has been implicated in regulating the TME, which is associated with reduced immune cell infiltration of B cells and CD8+ T cells, contributing to an immunosuppressive environment (90). HDAC6 also correlates with decreased expression of chemokines CXCL9, CXCL10, and CCL2, promoting an anti-inflammatory phenotype of TAMs.

Small molecule inhibitors of these enzymes have been shown to reprogram TAMs and synergize with ICIs in animal models of NSCLC (21). For example, HDAC6 inhibition combined with a PD-1 blockade demonstrated synergy, significantly inhibiting tumor growth and anti-tumor effects in LUAD, compared to HDAC6 inhibitor or PD-1 inhibitor monotherapy in C57BL/6 mouse models. Additionally, HDAC6 knockout significantly increases M1-type macrophage recruitment in LUAD and correlates with increased expression of CXCL9 and CXCL10, indicating its role in regulating macrophage polarization (90). Bag et al. (2022) demonstrated that the HDAC6 inhibitor ACY241, when combined with oxaliplatin, promoted a robust anti-tumor response, enhanced tumor-associated T cells’ functions, and prolonged survival in a pre-clinical mouse model of NSCLC, and was well tolerated without severe toxicity (91).

In TAMs, RIPK3 inhibits FAO by promoting the ROS/Caspase-1-mediated cleavage of PPARγ. When RIPK3 is downregulated, FAO is enhanced, promoting TAM polarization toward an M2-like tumor-promoting phenotype and enhanced immunosuppression. Decitabine, a DNA methyltransferase inhibitor, hypomethylates and re-expresses RIPK3, restoring ROS/Caspase-1/PPARγ-mediated suppression of FAO in TAMs, reversing the M2-like protumor phenotype of TAMs (7, 87, 92).

While clinical data are limited, early-phase trials are underway to evaluate safety and efficacy (3). In NSCLC, decitabine combined with aspirin significantly inhibits NSCLC growth and metastasis *in vivo* by targeting the β-catenin/STAT3 pathway, potentially affecting the immune microenvironment as a secondary effect (93).

### 3.5 MicroRNA -based reprogramming

MicroRNA (miRNA) plays a crucial role in regulating how macrophages polarize and function. They do this by modifying signalling pathways that control inflammatory and immunosuppressive responses. Several miRNAs, such as miR-155, miR-21, and miR-223, have been linked to TAM biology. These miRNAs influence pathways such as SOCS1, JAK/STAT1, and NF-κB signaling (68, 69). miR-155 supports M1-like polarization and enhances antitumor immunity. In contrast, miR-21 and miR-223 are associated with M2-like, immunosuppressive macrophage traits. Besides their intracellular roles, miRNAs derived from tumors can reshape TAMs, promoting immune evasion, metastasis, and tumor growth. Researchers have proposed disrupting the exchange of exosomal miRNA to interfere with tumor-macrophage communication and restore antitumor immunity (94).

Preclinical studies show that delivering miRNA mimics or antagomirs using lipid nanoparticles or designed exosomes can successfully reprogram TAMs towards inflammatory traits. Exosomal delivery of miRNAs such as miR-155, miR-125b-2, and miR-130 has been found to boost the expression of M1 markers while reducing M2-related gene activity. This leads to stronger antitumor immune responses and slower tumor growth across various cancer models (21, 94, 95). These results highlight the promise of using miRNA-based methods to change macrophage plasticity and gene expression in the tumor microenvironment. Building on this preclinical rationale, miRNA therapies have begun early clinical testing. The TargomiR program, which uses bacterially derived minicells to deliver a miR-16 mimic, is one of the most advanced platforms being tested in lung cancer and other thoracic tumors. Phase I studies showed that the treatment was generally well tolerated and engaged its target. However, significant clinical responses were limited among unselected patient groups. Overall, clinical data on miRNA therapies in NSCLC are still preliminary. Most studies report safety and pharmacodynamic effects rather than marked improvements in objective response rate (ORR), progression-free survival (PFS), or overall survival (OS) (96).

Translating miRNA-based TAM reprogramming into clinical practice faces significant challenges, including difficulties with delivering treatments specifically to tumors, unintended side effects, rapid clearance from the body, and limited uptake by macrophages. As a result, while miRNA agents can influence target pathways in living organisms, achieving lasting tumor shrinkage in advanced NSCLC remains a challenge. Current efforts focus on better delivery systems, targeting exosomes, selecting appropriate patients, and combining these agents with cytotoxic or immunomodulatory therapies to translate biological effects into significant clinical benefits (96).

### 3.6 Combination therapeutic approaches

Given the complex immunosuppressive role of TAMs in NSCLC, combination strategies that combine TAM-targeting agents with ICIs, chemotherapy, radiation, or epigenetic therapies have become the main clinical approach. Preclinical studies consistently show that modulating TAMs improves antitumor immunity when used with ICIs by reducing T-cell exclusion and restoring their cytotoxic effects across various tumor models, supporting clinical use (97). Early clinical trials have tested several combinations in advanced solid tumors, including NSCLC. In a phase 1b study, the anti-CSF1R antibody emactuzumab, combined with atezolizumab, provided pharmacodynamic evidence of TAM modulation and increased CD8+ T-cell infiltration; however, objective response rates were modest, especially in patients previously treated with immune checkpoint blockade (46). Similarly, the combination of AMG 820 with pembrolizumab showed acceptable tolerability but limited clinical efficacy, with an objective response rate of about 3% and identified hepatoxicity as a dose-limiting factor (45). These findings reveal a common gap between immunologic modulation and lasting clinical responses (**Figure 2**).

**Figure 2:**
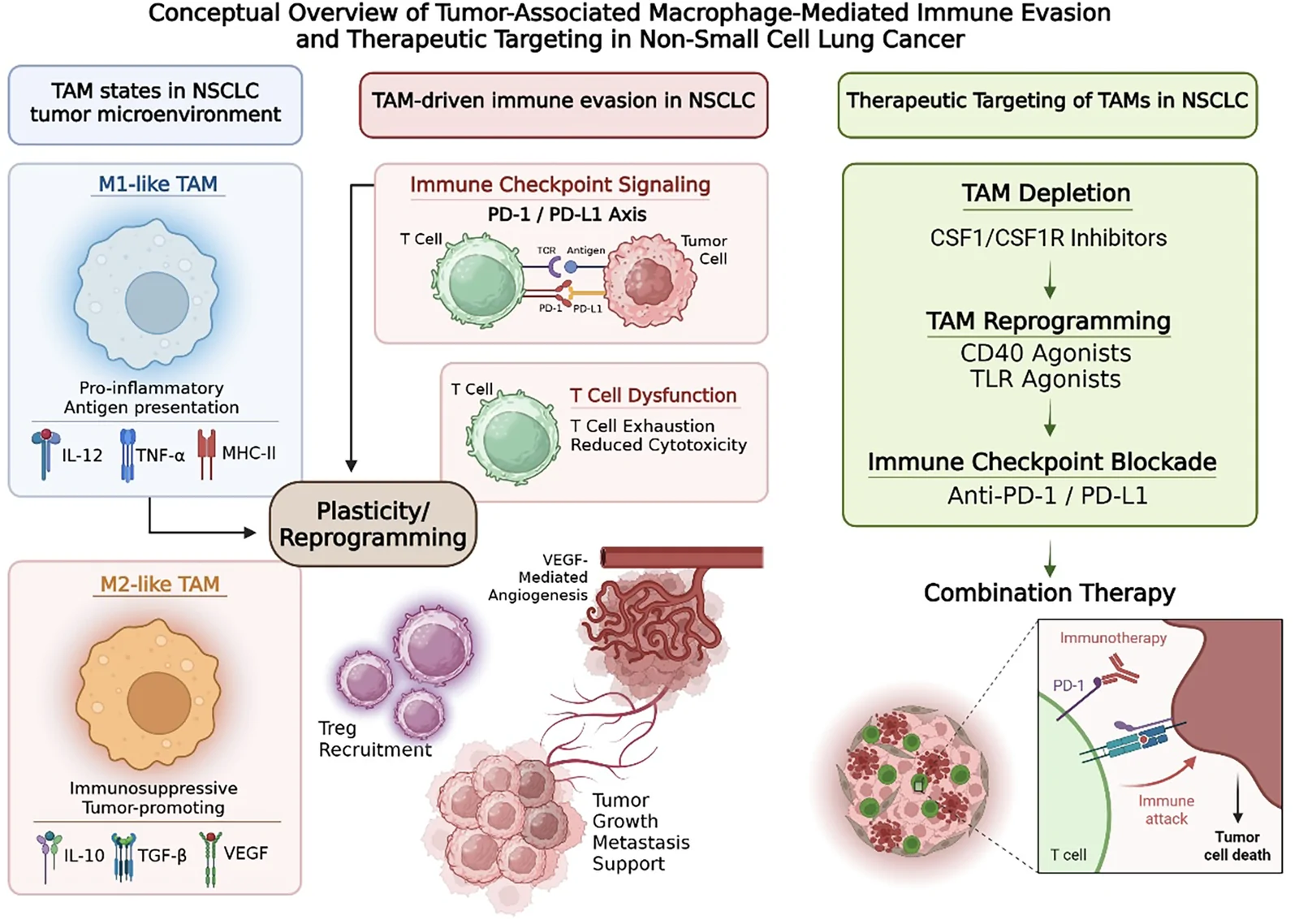
Conceptual overview of tumor-associated macrophage-mediated immune evasion and therapeutictargeting in non-small cell lung cancer. Created with BioRender.com Tumor-associated macrophages (TAMs) within the non-small cell lung cancer (NSCLC) tumor microenvironment exhibit functional plasticity, ranging from pro-inflammatory, anti-tumor states to immunosuppressive, tumor-promoting phenotypes. Immunosuppressive TAMs contribute to immune evasion through inhibition of cytotoxic T cell function, engagement of immune checkpoint pathways, recruitment of regulatory T cells, promotion of angiogenesis, and support of tumor growth and progression. Therapeutic strategies targeting TAMs include macrophage depletion, functional reprogramming toward anti-tumor states, and immune checkpoint blockade, often employed in combination to enhance anti-tumor immune responses in NSCLC.

Chemotherapy-based combinations offer another reason for synergy with TAM-targeted strategies. Drugs such as paclitaxel, cisplatin, and doxorubicin can cause immunogenic cell death, improve antigen presentation, and alter macrophage function within the tumor microenvironment (29). Paclitaxel activates Toll-like receptor 4 (TLR4) signalling, which triggers NF-kB and type I interferon pathways. This promotes a transition from M2-like to M1-like macrophage states and makes tumors more sensitive to ICIs (98, 99). Chemotherapeutic agents also boost the expression of co-stimulatory molecules such as CD80 while lowering inhibitory checkpoint ligand expression, further encouraging immune activation (98, 100). Clinically, a phase II study that combined ipilimumab with paclitaxel and carboplatin in previously untreated NSCLC showed that phased administration allowed chemotherapy-induced antigen release to prime antitumor immune response (101, 102). In addition to direct TAM-targeting agents, epigenetic modulators present an indirect but valuable approach for reshaping suppressive myeloid compartments. The combination of the HDAC inhibitor entinostat with pembrolizumab displayed some activity in NSCLC patients who had previously received ICIs. This shows that epigenetic modulation of myeloid cells can enhance immune checkpoint blockade, even though it is not specific to macrophages (57, 58).

Despite strong reasons from preclinical studies, combination strategies frequently encounter clinical challenges. These include overlapping toxicities, especially hepatotoxicity seen with CSF1R inhibitors combined with ICIs, adaptive resistance due to compensatory recruitment of myeloid-derived suppressor cells and neutrophils, and the persistence of TAM subsets such as SPP1+ and TREM2+ macrophages, which are insensitive to the targeted pathway. Even when TAM modulation briefly increases T-cell infiltration, tumors often revert to immunosuppression through alternative chemokine axes (e.g., CCL2-CCR2, CXCL8-CXCR2) or metabolic pathways involving adenosine and lactate. Therefore, achieving durable benefits will likely require combination strategies that target multiple pathways, biomarker-guided patients selection, and dosing schedules that consider potential toxicities (46, 58, 98).

### 3.7 Engineered macrophages for drug delivery

Macrophages have natural chemotactic capabilities, enabling them to home to tumor sites, making them a potential target for cell-based drug delivery. They secrete cytokines, chemokines, and growth factors, such as CSF1, VEGFA, CCL2, and CCL18, which mediate the recruitment of monocytes/macrophages to the tumor, as seen in various tumor models (103, 104). Engineered macrophages can deliver cytotoxic agents, cytokines, or nanoparticles to the TME. For example, genetically engineered macrophages (GEMs) express and secrete interleukin 12 (IL-12), delivering their payload to the TIME and stimulating anti-tumor responses (105, 106).

Chimeric antigen receptor (CAR)-Macrophages (CAR-M) is a breakthrough strategy that involves the GEMs matching tumor-specific receptors. CAR-M possesses anti-tumor properties, activating immune responses and targeting tumor antigens, such as CD19, CD22, and HER2 (106, 107). The CAR-M cells have also proven to show strong phagocytosis, antigen presentation, and TME remodelling in preclinical models of NSCLC (23, 82). In murine tumor cell lines that overexpress HER2, murine CAR-M demonstrated antitumor activity by remodeling the immunosuppressive TIME and inducing CD8+ and CD4+ T cell infiltration in solid tumors (108). CAR-M therapy can be extended to other solid tumors, including NSCLC. A novel CAR-M has been developed using anti-MUC1 scFv (mucin 1 tumor antigen) targeting lung tumors. It differentiates into a pro-inflammatory M1 phenotype, expressing TNF-α, IL-8, and IL-1β cytokines, and exhibits tumor-restricted phagocytosis (109).

Exosomes are also equipped to deliver miRNAs or drugs therapeutically, relying on endogenous capabilities of macrophage-derived exosomes. Nevertheless, limitations such as off-target effects, immune activation, and scalability remain obstacles to clinical translation (79).

## 4 Challenges and limitations of TAM-targeted therapies in NSCLC

Although TAM-targeted therapies hold promise, clinical translation remains a challenge. Some of the limiting factors include the limited generalizability of TAM subsets, the deficiency of predictive markers, and possible off-target effects. TAMs are highly heterogeneous, with distinct subpopulations that depend on tumor type, stage, and TIME cues. Their roles can range from promoting to suppressing the tumor, varying between different cancers and even among patients with the same cancer, which complicates the isolation of TAMs and effective therapy design (110–112).

Single-cell RNA sequencing, spatial transcriptomics, and multiplex imaging are emerging technologies to determine TAM signatures that predict response to therapy (4, 68). Moreover, circulating cytokines or exosomal content can be dynamically monitored as non-invasive biomarkers of macrophages reprogramming efficacy (4). For example, serum cytokines can serve as prognostic markers for NSCLC patients undergoing anti-PD-1 blockade therapy, with IL-6 being associated with poor prognosis. IL-6 affects PD-1/PD-L1 interactions in TAMs by reducing IFNγ production. Therefore, targeting IL-6 in combination with PD-1 blockade can offer a therapeutic advantage (113). Ongoing trials are evaluating tailored therapies of TME profile and optimizing combinatorial therapies, yet clinical data are still limited (22, 114). TAMs play a central role in controlling immune environments in NSCLC and establishing immunosuppression and resistance against ICIs. Therapeutic reprogramming of TAMs through molecular, metabolic, and genetic intervention to shift the tumor cells of NSCLC into an immunologically competent microenvironment holds great promise.

### 4.1 Systemic toxicities

Although therapies targeting TAMs are generally considered well tolerated—especially when administered locally or through nanomedicine approaches that specifically deliver drugs to TAMs while minimizing systemic toxicity—there are challenges related to toxicity and adverse events. These issues can be dose-dependent, particularly when used in combination with other treatments, which constrain dose intensity and combinations, leading to failure in NSCLC. There are also concerns with macrophage delivery systems related to pro-inflammatory immune system activation and systemic toxicities due to on-target off-tumor effects (115).

CSF1R inhibitors can reprogram macrophages but also disrupt Kupffer cells, which can result in elevated AST/ALT levels and other hepatocellular enzymes caused by decreased clearance, leading to transient liver-related adverse events. Additionally, CSF-1/CSF-1R inhibitors are associated with hematological toxicities, particularly anemia, as seen with agents like pexidartinib, emactuzumab, lacnotuzumab, and AMG 820. Adverse events seen in these agents are also associated with skin toxicities, edema, and gastrointestinal events such as nausea, vomiting, and diarrhea (116, 117).

Preclinical work also indicates that the activation status of macrophages plays a crucial role in mediating liver damage. For example, the administration of a CD40 agonist before chemotherapy can lead to lethal hepatotoxicity, which significantly activates macrophages. Inhibition of macrophage activation using CSF-1R blockade abolishes liver toxicity and inhibits tumor growth (118).

Similarly, CD47 blockade is associated with adverse events, primarily on-target transient anemia, resulting from the phagocytosis of RBCs. Other adverse events associated with CD47 are observed, including infusion reactions, fatigue, headaches, fever, neutropenia, thrombocytopenia, and hyperbilirubinemia. Combinations with other cytotoxic agents can also lead to enhanced pro-phagocytic signals on cells, resulting in potential enhanced toxicities (119, 120).

### 4.2 Drug delivery challenges in hypoxic and dense NSCLC

In addition to systemic toxicity, the hypoxic and dense TME in NSCLC presents a major physical and immunological barrier to drug delivery of therapies targeting TAMs and overcoming resistance. The NSCLC TME is characterized by spatial niches that form a complex and dynamic system, influencing each other and interacting with treatments. Hypoxia in tumors leads to alterations that promote an immunosuppressive environment, contributing to tumor metastasis, immune escape, drug resistance, and poor outcomes (121, 122). Hypoxia activates cancer-associated fibroblasts (CAFs), resulting in the production of ECM and the formation of fibrotic niches. This abnormal, disorganized, and leaky vasculature, along with a dense ECM, exacerbates hypoxia and impairs blood flow. Consequently, this acts as a barrier to infiltrating immune cells and upregulates HIF expression and immune checkpoints on cancer cells. As a result, the TME becomes immunologically “cold”, dominated by immunosuppressive cells such as Tregs, MDSCs, and M2 polarization, collectively leading to ICIs resistance. In preclinical models of NSCLC, the upregulation of HIF-1α is associated with resistance to EGFR TKIs. This complicates targeting the NSCLC TME, as treatments may unintentionally aggravate immune-related toxicities, trigger resistance mechanisms, or exert a protumorigenic effect (122).

### 4.3 Recruitment of suppressive myeloid cells

A common finding in clinical and preclinical studies is that removing or reprogramming one type of suppressive myeloid cell often leads to the recruitment of others, especially MDSCs and neutrophils. These cells quickly restore an immunosuppressive environment. Blocking pathways that support macrophages, such as CSF1/CSF1R, change the chemokine and growth factor signals in the TIME and stromal areas, including CAFs. This change causes an increase in alternative signals, such as CXCL1/8 and CCL2, which draw in PMN-MDSCs or CCR2+ monocytes. Overall, this results in a temporary increase in T-cell infiltration, but as new suppressive myeloid populations appear, this effect diminishes. This explains why simply removing TAMs often does not lead to long-lasting clinical improvements (112, 123). Therefore, interventions that target TAMs usually need to combine treatments that also address other compensatory pathways, such as blocking CSF1R along with CXCR2 or CCR2, or use strategies that anticipate rebounds in recruitment.

### 4.4 Macrophage plasticity

Macrophages are some of the most adaptable immune cells in the TIME. They can change their function and gene expression quickly in response to local signals, such as cytokines, metabolites, and contact with other cells. When exposed to IFN-γ or TLR signals, macrophages may shift to inflammatory states that present antigens, marked by CXCL9. In contrast, signals such as IL-4, IL-13, TGF-β, lactate, and adenosine push them toward M2-like status, focused on tissue repair or lipid handling. This adaptability offers opportunities for reprogramming, but it also means that macrophages can quickly return to their original states if the microenvironment does not change significantly. Recent studies at the single-cell and metabolic level show that metabolic signals, such as low oxygen, lactate buildup, and lipid levels, are closely linked to the macrophage’s gene expression and function. Shifting metabolism back toward an inflammatory program is necessary but often insufficient unless the upstream hypoxic/metabolic drivers are addressed (5, 124). The plasticity of macrophages requires multi-faceted strategies that address both signaling and metabolic factors.

### 4.5 Reprogramming instability and practical consequences

Even when TAMs can be temporarily changed to an M1-like state in early clinical trials, the instability of this reprogramming limits the duration of the benefits. The reasons include ongoing immunosuppressive factors in the TIME, such as IL-10, TGF-β, and PGE2, metabolic feedback such as lactate-driven changes to histones that support M2 programs, and the continuous influx of new TAMs from monocytes that refill suppressive niches (5, 125). Clinically, this instability is seen as temporary changes in drug effects, like a short-term influx of CD8^+^ T cells or marker changes, without lasting benefits or improved survival in many early-stage trials.

To address these challenges, new strategies are emerging including: (a) combinations that target both TAMs and potential compensatory pathways, such as blocking CSF1R and CXCR2 or CCR2, (b) metabolic interventions to counteract lactate and adenosine signals, which helps stabilize inflammatory responses, and (c) therapies that take spatial context into account, targeting specific areas like hypoxic or SPP1-positive regions compared to perivascular areas. The field also needs reliable, dynamic biomarkers, such as patterns of spatial CXCL9 and SPP1, and circulating levels of chemokines and MDSC signatures. These would help identify patients and detect early compensatory responses, allowing for timely adjustments to therapy (112, 123). Without these combined, multi-pronged strategies focused on biomarkers, efforts to target TAMs may lead to only temporary immune responses rather than lasting clinical improvements.

## 5 Conclusion and future directions

Despite progress in the treatment of NSCLC, the clinical translation of these therapies remains limited due to challenges with drug delivery, systemic toxicities, immunosuppression, macrophage plasticity, and heterogeneity. Future directions for TAM-targeted therapy in NSCLC depend on overcoming limitations in delivery efficiency, selectivity, and patient stratification. Optimizing delivery systems that can penetrate the tumor microenvironment and selectively manipulate TAM phenotypes is important. Currently, nanoparticles, such as liposomes, exosomes, and hybrid nanoparticles, are being actively evaluated for their effectiveness in precise drug coating, release, and delivery. Patient variability in TAMs and immune cell dynamics in TIME, as well as their responsiveness to treatment, poses significant challenges. However, there is a growing interest in non-invasive imaging and monitoring of multiple biomarkers. Utilizing multi-omics biomarkers, including transcriptomics, epigenetics, and spatial signatures of TAM populations, is crucial for guiding patient selection, profiling the TIME, and predicting treatment outcomes. This supports precision therapy plans, especially in the context of immunotherapy (4, 21, 126–128). With continuous studies and clinical trials, targeting TAMs may become a cornerstone of NSCLC immunotherapy.
